# Hyperfine interaction in atomically thin transition metal dichalcogenides†

**DOI:** 10.1039/c8na00360b

**Published:** 2019-05-13

**Authors:** Ivan D. Avdeev, Dmitry S. Smirnov

**Affiliations:** Ioffe Institute 194021 St. Petersburg Russia smirnov@mail.ioffe.ru

## Abstract

The spin dynamics of localized charge carriers is mainly driven by hyperfine interaction with nuclear spins. Here we develop a theory of hyperfine interaction in transition metal dichalcogenide monolayers. Using group representation theory and the tight binding model we derive effective Hamiltonians of the intervalley hyperfine interaction in the conduction and valence bands. The spin–valley locking and pronounced spin–orbit splitting lead to a specific form of hyperfine interaction, which we call “helical”. We also demonstrate that the hyperfine interaction is noncollinear for chalcogen atoms in the general case. At the same time in the upper valence band the hyperfine interaction is purely of the Ising type, which suggests that the spin–valley polarization of localized holes in transition metal dichalcogenide monolayers can be conserved for a particularly long time.

## Abstract 2

A theory of spin–valley dynamics in transition metal dichalcogenide monolayers induced by hyperfine interaction is developed.

## Graphical abstract

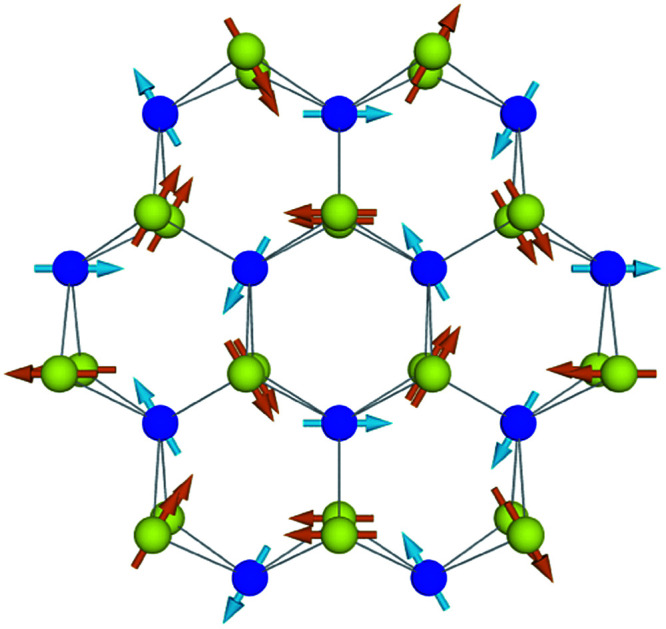

## 1. Introduction

Atomically thin transition metal dichalcogenides (TMDs), MX_2_ with M being a transition metal (Mo and W) and X being a chalcogen (S, Se, and T), represent a new generation of truly two-dimensional structures.^1^ TMD monolayers (MLs) have a direct band gap at the two inequivalent ***K***_+_ and ***K***_−_ points of the Brillouin zone.^2,3^ The conduction and the valence bands at these points are split by the pronounced spin–orbit interaction, which leads to the so-called spin–valley locking and valley dependent optical selection rules.^4,5^ The large exciton binding energy of about 0.5 eV (ref. 1) in TMD MLs makes it possible to manipulate spin–valley polarization up to room temperature.^6–8^ These unique properties can be useful for a broad range of applications.^1,9,10^

Particularly promising for optoelectronic devices are zero-dimensional systems, like quantum dots based on TMD MLs.^11,12^ In principle any disorder in 2D structures leads to the localization of charge carriers.^13^ In practice TMD ML quantum dots can be made by chemical exfoliation^14–16^ and lithographic nanopatterning^17^ or charge carriers can be trapped by wrinkles,^18^ homojunctions,^19^ or defects.^20–22^

The spin–valley polarization lifetime for excitons is unavoidably limited by the exciton lifetime being in a few picosecond range.^23^ But this limitation is released for resident charge carriers. Their polarization can be preserved for a few nanoseconds in MoS_2_,^24–26^ and even longer in WSe_2_.^27–29^ For free charge carriers the polarization relaxation is related to the spin–orbit interaction, which can be suppressed by localization. In this case the dominant role in the spin and valley dynamics is played by the hyperfine interaction with the host lattice nuclear spins.^30^

In TMD MLs, in contrast to many other materials, the electron nuclear spin flips within a valley are suppressed by spin–orbit splitting, which is about 10^4^ times larger than the hyperfine interaction. At the same time the intervalley spin flips should be accompanied by the transfer of the large momentum, equal to ***K***_+_ − ***K***_−_. This is, however, easily possible because of the very short range nature of the hyperfine interaction. For this reason in our work we will focus only on the intervalley hyperfine interaction and neglect spin flips in one valley.

Remarkably, the hyperfine interaction has the same relativistic origin as the spin–orbit interaction, so one can expect that it is also strong in TMD MLs. At the same time, the related effects in TMD MLs stay essentially unexplored.^31,32^ In this work we derive the Hamiltonian of the hyperfine interaction from a rigorous symmetry analysis and corroborate our results using the tight binding model.

We show that the low symmetry of TMD MLs allows for the noncollinear hyperfine interaction,^33,34^ so the nuclear spins can be flipped without the need to flip the valley pseudospin. This effect was previously observed in GaAs based quantum dots, where it manifested itself as a dragging of the quantum dot resonance frequency.^35^ Our calculations show that this effect is about two orders of magnitude stronger in TMD ML quantum dots.

The locking of spin and valley degrees of freedom also brings specifics to the hyperfine interaction. In this work we demonstrate that it leads to a “helical” structure of the interaction of the valley pseudospin with nuclei, which means that the components of the hyperfine interaction Hamiltonian are periodically modulated in space. This effect manifests itself in the dynamic nuclear spin polarization and formation of the nuclear spin polaron, which also have a helical structure.

Previous studies of spin relaxation in TMD ML quantum dots predicted the divergence of the polarization relaxation time in zero magnetic field.^36^ Physically the lifetime of spin–valley polarization in zero magnetic field is limited by the hyperfine interaction. We show that for electrons the interaction with nuclei leads to spin dephasing on the timescale of tens of nanoseconds. For holes, in contrast to previous misleading studies,^31^ we show that the hyperfine interaction in the upper subband is of the Ising type. So the polarization relaxation of holes is parametrically longer than that of electrons.

The paper is organized as follows. In Sec. 2 we perform the symmetry analysis of the hyperfine interaction. Then, in Sec. 3, using the tight binding model we calculate the components of the hyperfine interaction tensors. The main physical results are derived and discussed in Sec. 4. Finally, the conclusions are given in Sec. 5.

## 2. Symmetry analysis

**Fig. 1:**
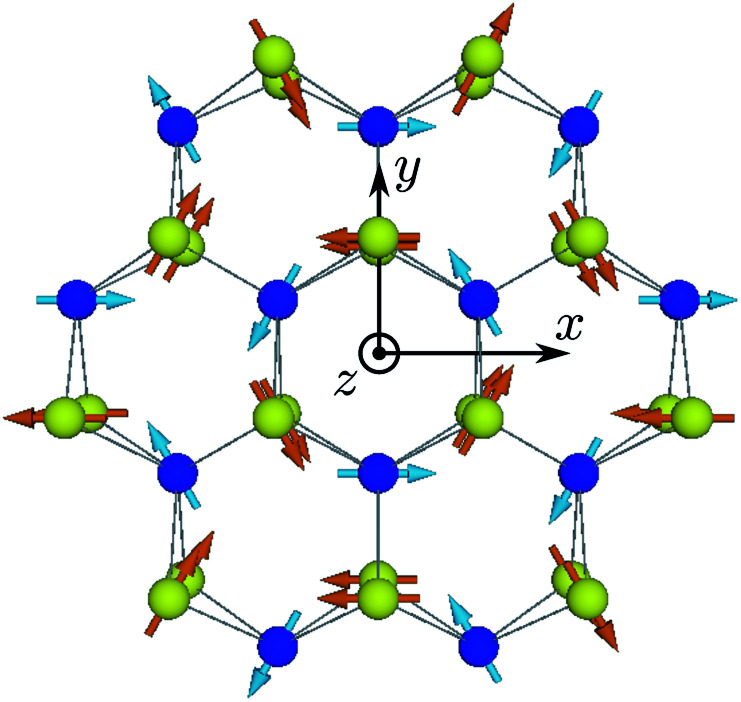
A part of TMD ML and the coordinate frame. The blue and yellow balls show the metal and chalcogen atoms, respectively, with arrows corresponding to the nuclear spins. The orientation of nuclear spins corresponds to the dynamic nuclear polarization induced by the valley pseudospin polarized along the *x* axis.

A single TMD ML has a honeycomb lattice with one transition metal atom and two chalcogen atoms in the unit cell; see the ESI† for more details. A few unit cells of TMD MLs are shown in Fig. 1, where the metal and chalcogen atoms are represented by blue and yellow balls, respectively. The metal atoms lie within the ML plane, (*xy*), while the chalcogens are shifted along the *z* axis in the opposite directions from the ML plane.

We chose the origin of the coordinate frame at the center of the hexagon, formed by the metal and chalcogen atoms,^1,2^ as shown in Fig. 1. We also choose the *y* axis to be oriented towards the nearest pair of chalcogen atoms. We note that caution should be taken regarding the choice of the coordinate frame origin and orientation of the axes, when comparing with the results of some other authors.^3,37^

The point symmetry of the TMD ML is *D*_3h_. This group consists of a horizontal (lateral) reflection plane *σ*_h_‖(*xy*), three fold rotation axis *C*_3_‖*z*, three vertical reflection planes 3*σ*_v_, three in-plane two fold rotation axes 
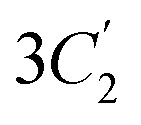
 (including the *y* axis), and the combinations *S*_3_ = *σ*_h_*C*_3_. In total, there are 12 symmetry operations including identity.

The valence and conduction band extrema are located at the two inequivalent ***K***_±_ points of the Brillouin zone, as described in the Introduction; also see the ESI† for more details. The wave vector point symmetry in these valleys is *C*_3h_, which is a subgroup of *D*_3h_ lacking all the elements interchanging the ***K***_+_ and ***K***_−_ valleys.

**Fig. 2:**
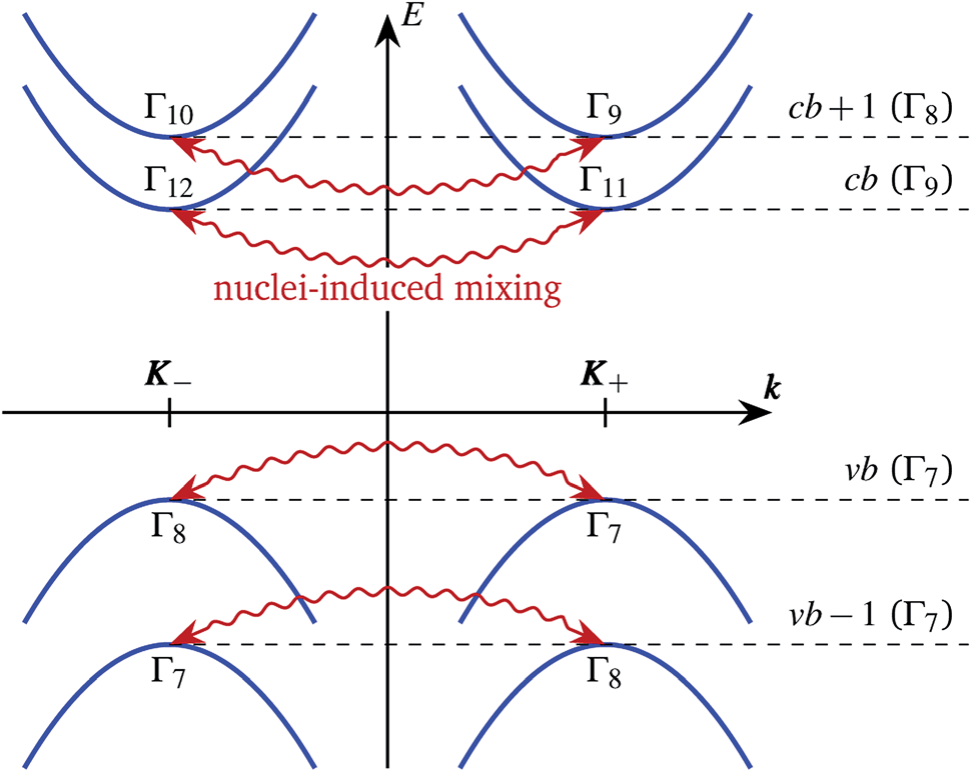
Schematics of the band structure. The red wavy arrows show the intervalley hyperfine interaction. Representations of the electronic states in the *C*_3h_ group are shown for each valley and each band. On the right the representations of pairs of energy degenerate states in the *D*_3h_ group are given.

All the irreducible representations of the *C*_3h_ group are one dimensional, so all electronic states in ***K***_±_ valleys are nondegenerate, as shown in the band diagram in Fig. 2. However, the two valleys are related by the time reversal symmetry and their energies coincide, in agreement with the Kramers theorem.

We focus our attention on the four (sub)bands in the vicinity of the band gap, which we label by the index *m* = *cb* + 1,*cb*,*vb*,*vb* − 1, as shown in Fig. 2. The electron wave function in the *K*_±_ valley in the *m*th band has the general form

1 ***Ψ***^(*m*)^_±_(***r***) = e^i**K**_±_***r***^*u*^(*m*)^_±_(***r***),

where *u*^(*m*)^_±_(***r***) is the Bloch amplitude (a spinor). Note that the functions ***Ψ***^(*m*)^_+_(***r***) and ***Ψ***^(*m*)^_−_(***r***) are related by the time reversal symmetry and *σ*_v_ reflections.

The nuclear spins weakly break the translation symmetry of the structure and lead to the splitting and mixing of the states in ***K***_+_ and ***K***_−_ valleys. The strength of the hyperfine interaction with the nuclear spins is usually of the order of 1 μeV (ref. 30 and 31). This is much smaller than the spin–orbit splittings of the conduction and valence bands in TMD MLs, which are of the order of a few tens of meV and a few hundreds of meV, respectively.^2,38^ Therefore, the hyperfine interaction can mix only the states with the same energy, *i.e.* in the same band, but in different valleys, as shown by the wavy arrows in Fig. 2.

The two states in *K*_+_ and *K*_−_ valleys can be interpreted as a valley qubit.^39–41^ We introduce the valley pseudospin matrices ***τ̂*** = (*τ̂*_*x*_,*τ̂*_*y*_,*τ̂*_*z*_), see the ESI†, so that *K*_±_ states correspond to *τ*_*z*_ = ±1/2, respectively. We note that due to the difference of the Bloch wave vectors ***K***_+_ and ***K***_−_, spin is not a good quantum number, so the hyperfine interaction Hamiltonian should be written in terms of the valley pseudospin ***τ***. Taking into account the form of the electron wave functions, eqn (1), the hyperfine interaction Hamiltonian in the *m*th band is2
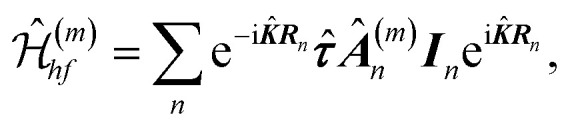
where *n* is the number of the nuclei with the spins ***I***_*n*_ and the two dimensional coordinates ***R***_*n*_ = (*R*_*n*,*x*_, *R*_*n*,*y*_), ***K̂*** = 2***K***_+_*τ̂*_*z*_ is the momentum operator and *Â*^(*m*)^_*n*_ is the tensor of the hyperfine interaction. Here we explicitly wrote the two operators (or matrix exponents) e^±i***K̂R***_*n*_^, which account for the relative spatial phase shift between ***Ψ***^(*m*)^_+_(***r***) and ***Ψ***^(*m*)^_−_(***r***). As a result, the tensors *Â*^(*m*)^_*n*_ are independent of the position of the elementary cell in the TMD ML. Moreover, the hyperfine interaction tensors for each pair of chalcogen atoms in the same unit cell are related by *σ*_h_ reflection, *i.e.* they are linearly dependent. As a result there are only two independent hyperfine interaction tensors in each band: one with the metal and one with the chalcogen atoms.

Further symmetry analysis allows us to find the restrictions on the form of the hyperfine interaction tensors. It is most convenient to start the analysis from the *C*_3h_ group of the wave vector, and then consider the raise of the symmetry up to *D*_3h_, the point symmetry of the structure.

The representations corresponding to the standard choice of the origin of the coordinate frame at the center of the hexagon are well established.^1,42^ But to analyze the hyperfine interaction tensor with the *n*th nucleus, it is convenient to move the origin of the coordinate frame towards the corresponding nucleus by the two-dimensional vector ***R***_*n*_. Upon this nontrivial translation, the irreducible representations of the wavefunctions ***Ψ***^(*m*)^_±_ change. In the ESI† we show that for each symmetry operation *g* of the wave vector group *C*_3h_ the matrix of the representation should be multiplied by

3 e^−i***K***_±_(***R***_*n*_−*g****R***_*n*_)^.

The sets of these factors form a representation, corresponding to the function4
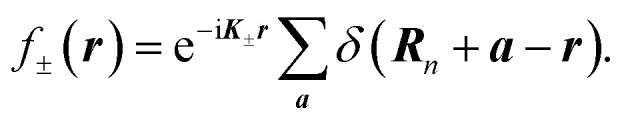
Here the sum runs over all translation vectors ***a*** and *δ*(***r***) is the Dirac *δ*-function. The functions *f*_±_ transform according to Γ_2,3_ (Γ_3,2_) representations of the *C*_3h_ point symmetry group for the *n*th atom being a metal (chalcogen), respectively. Therefore, the representations of the wavefunctions for the two different origins of the coordinate frame are related simply by multiplication with the representation Γ_2_ or Γ_3_.

In Table 1 we present the irreducible representations of the electronic states in the *C*_3h_ group in the bands under study. The representations corresponding to the shifted origin of the coordinate frame can be calculated using the multiplication rules for the *C*_3h_ group.^43^ The two representations, corresponding to the two valleys in the same band, are always conjugate, in agreement with the time reversal symmetry.

**Table 1:** Spinor irreducible representations of the electronic states in the *C*_3h_ group for the ***K***_±_ valleys for different choices of the coordinate frame origin: in the center of the hexagon (O), at the metal atom (M), and between neighboring chalcogen atoms (X)^a^

Band	O		M		X	
	***K***_±_	***K***_−_	***K***_+_	***K***_−_	***K***_+_	***K***_−_
*cb* + 1	Γ_9_	Γ_10_	Γ_8_	Γ_7_	Γ_12_	Γ_11_
*cb*	Γ_11_	Γ_12_	Γ_7_	Γ_8_	Γ_10_	Γ_9_
*vb*	Γ_7_	Γ_8_	Γ_10_	Γ_9_	Γ_11_	Γ_12_
*vb* − 1	Γ_8_	Γ_7_	Γ_12_	Γ_11_	Γ_9_	Γ_10_

a The order of bands corresponds to the molybdenum based structures.

Now we consider the point symmetry group *D*_3h_ of the TMD ML. The conjugate representations of the *C*_3h_ group join in the *D*_3h_ group in pairs as follows (see the ESI†):

5a {Γ_8_(*C*_3h_), Γ_7_(*C*_3h_)} → Γ_7_(*D*_3h_),

5b {Γ_10_(*C*_3h_), Γ_9_(*C*_3h_)} → Γ_8_(*D*_3h_),

5c {Γ_11_(*C*_3h_), Γ_12_(*C*_3h_)} → Γ_9_(*D*_3h_).

The order of the representations in the curly brackets corresponds to the standard basis of the representation of the *D*_3h_ group.^43^ This rule, together with Table 1, allows one to find the irreducible representations of the pairs of the wavefunctions ***Ψ***^(*m*)^_±_ in the *D*_3h_ group with the coordinate frame origin located at ***R***_*n*_. The results are listed in the second columns of Tables 2 and 3. One can see that it can be either Γ_7_, or Γ_8_, or Γ_9_. For the rest of the paper all irreducible representations are given for the *D*_3h_ group.

**Table 2:** Relationships between the components of the tensors of the hyperfine interaction with the metal atoms^a^

Band	irrep (M)	*A* ^M^_*xx*_/*A*^M^	*A* ^M^_*yy*_/*A*^M^	*A* ^M^_*zz*_/*A*^M^
*cb* + 1	Γ_7_	2/7	−2/7	−4/7
*cb*	Γ_7_	2/7	2/7	4/7
*vb*	Γ_8_	0	0	24/7
*Vb −* 1	Γ_9_	0	0	32/7

a Additionally the second column shows the irreducible representations in the *D*_3h_ group with the center of transformations at a metal atom.

**Table 3:** Relationships between the components of the tensors of the hyperfine interaction with the chalcogen atoms^a^

Band	irrep (X)	*A* ^X^_*xx*_/*A*^X^	*A* ^X^_*yy*_/*A*^X^	*A* ^X^_*zz*_/*A*^X^
*cb* + 1	Γ_9_	0	0	−8/5
*cb*	Γ_8_	−6/5	6/5	−12/5
*vb*	Γ_9_	0	0	8/5
*vb −* 1	Γ_8_	−6/5	−6/5	12/5

a Notations are the same as in Table 2.

The irreducible representations corresponding to the components of the valley pseudospin ***τ̂*** can be found from the decomposition of the squares of the self-conjugate representations found above. The multiplication rules are^43^

6a Γ_7_ ⊗ Γ_7_ = Γ_8_ ⊗ Γ_8_ = Γ_1_ ⊕ Γ_2_ ⊕ Γ_5_,

6b Γ_9_ ⊗ Γ_9_ = Γ_1_ ⊕ Γ_2_ ⊕ Γ_3_ ⊕ Γ_4_.

Specifically, in the ESI† we show that for the representations Γ_7_ and Γ_8_ the valley pseudospin component *τ̂*_*z*_ transforms according to Γ_2_, while *τ̂*_*x*_ and *τ̂*_*y*_ form the basis of the representation Γ_5_. For the representation Γ_9_ we find that *τ̂*_*z*_ again transforms according to Γ_2_, while *τ̂*_*x*_ and *τ̂*_*y*_ form the bases of the representations Γ_4_ and Γ_3_, respectively.

Now let us turn to the classification of the nuclear spin components. We recall that we perform the symmetry analysis in the point symmetry group *D*_3h_ with the center of transformations at ***R***_*n*_. The nuclear spin of a metal atom, ***I***_M_, is a pseudovector, and its components transform according to Γ_2_ (*I*_M,*z*_) and Γ_5_ (∓*I*_M,*x*_ − i*I*_M,*y*_) representations. The two chalcogen atoms at the two-dimensional coordinate ***R***_*n*_ (in the same unit cell) exchange their places under the reflection in the horizontal plane *σ*_h_. So we introduce the linear combinations of their spins ***I***_X_ = (***I***^up^ + ***I***^down^)/2 and Δ***I*** = ***I***^up^ − ***I***^down^, where the superscripts “up” and “down” refer to the spins of the atoms above and below the (*xy*) plane. Under the reflection *σ*_h_ the components of ***I***_X_ transform in the same way as those of ***I***_M_, while the components of Δ***I*** additionally change the sign. Under the reflection in the vertical plane *σ*_*v*_ the components of both ***I***_X_ and Δ***I*** transform in the same way as those of ***I***_M_. As a result the components *I*_X,*z*_ and ∓*I*_X,*x*_ − i*I*_X,*y*_ belong to Γ_2_ and Γ_5_ representations, respectively; the component Δ*I*_*z*_ belongs to Γ_3_, while Δ*I*_*x*_ and Δ*I*_*y*_, form the basis of the representation Γ_6_.

Now the symmetry analysis of the hyperfine interaction becomes straightforward. According to the method of invariants the coupling is allowed only between the components of ***τ̂*** and ***I***, which transform according to the same irreducible representation. For the representations Γ_7_ and Γ_8_ corresponding to the coordinate ***R***_*n*_ the hyperfine interaction Hamiltonian has the form:7
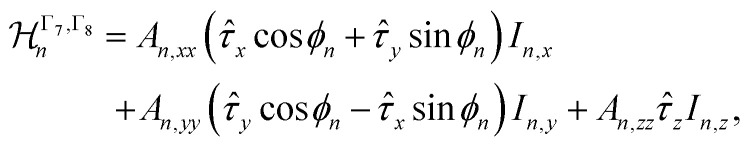
where we took into account the phase factors in eqn (2)*ϕ*_*n*_ = 2*KR*_*n*,*x*_ with *K* = |***K***_±_|. The absolute values of the in-plane hyperfine coupling constants are equal: |*A*_*n*,*xx*_| = |*A*_*n*,*yy*_|, while their signs can be opposite or the same depending on the representations of the electronic states in *K*_+_ and *K*_−_ valleys in the *C*_3h_ group. Below we focus on the molybdenum based structures, as for the tungsten based ones the results are the same, except for the inversion of the bands *cb* and *cb* + 1. Thus, *A*_*xx*_ = *A*_*yy*_ for Mo atoms in the band *cb* and for chalcogen atoms in the band *vb* − 1. By contrast, for Mo atoms in the bands *cb* + 1 and *vb* and for chalcogen atoms in the band *cb* one has *A*_*yy*_ = − *A*_*xx*_. These results are summarized in Tables 2 and 3.

In the case of the representation Γ_9_ the Hamiltonian has the form8
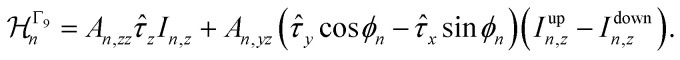
Here in the second line ***I***^up,down^_*n*_ are the spins of chalcogen atoms above and below the (*xy*) plane and this term is absent for Mo atoms. The Hamiltonian of this type is relevant for Mo atoms in the band *vb* − 1 and for the chalcogen atoms in the bands *vb* and *cb* + 1.

One can see that the component *A*_*zz*_ is symmetry allowed for all atoms in all bands. This means that the nuclear spin polarization along *z* creates a longitudinal Overhauser field and lifts the Kramers degeneracy of the bands, similarly to an external longitudinal magnetic field. Similarly the valley polarization along the *z* direction creates a Knight field perpendicular to the monolayer plane, which acts on the nuclei. The difference between *A*_*zz*_ in the pairs of bands *cb* and *cb* + 1, and *vb* and *vb* + 1 is analogous to the longitudinal spin *g* factor of the charge carriers.^44,45^

The in-plane hyperfine coupling is allowed only for certain bands and it depends on the coordinates of the nuclei. The phase *ϕ*_*n*_ in eqn (7) and (8) effectively describes the rotations of nuclear spins, so we call this hyperfine interaction helical. We stress that the helical hyperfine interaction is a direct consequence of the spin–valley locking inherent to TMD MLs. Note also that the second term in eqn (8) describes the noncollinear hyperfine interaction. We discuss the corresponding physical effects in Sec. 4.

## 3. Tight binding model

The hyperfine interaction of the charge carriers with the host lattice nuclei is a short range interaction,^31,46^ so it can be studied quantitatively using the tight binding approximation. This is done in this section.

The microscopic Hamiltonian of the hyperfine interaction with the nuclei has the form9
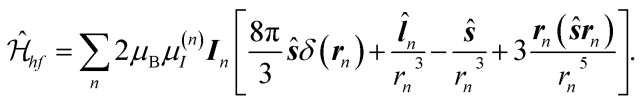
Here *μ*_B_ is the Bohr magneton, *μ*^(*n*)^_*I*_, ***r***_*n*_ is the electron distance to the *n*th nucleus (being a three dimensional vector), *l̂_n_* = −*i*[***r***_*n*_ × **∇**] is the electron angular momentum, and ***s*** is the electron spin operator. The first term in square brackets in eqn (9) describes the Fermi contact interaction, which vanishes for all atomic orbitals except for the *s* ones. The other terms describe the magnetic dipole–dipole interaction.

For the sake of simplicity, we limit ourselves to d orbitals at the metal atoms (thus neglecting s orbitals^47^) and p orbitals at the chalcogen atoms.^2,37^ Bearing in mind the irreducible representations summarized in Table 1, one finds the form of the Bloch amplitudes in the tight-binding model.^2,37^ We neglect the mixing between spin-up and spin-down states and obtain10a
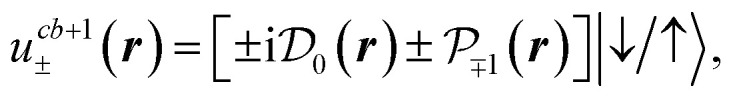
10b
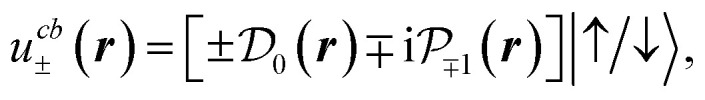
10c
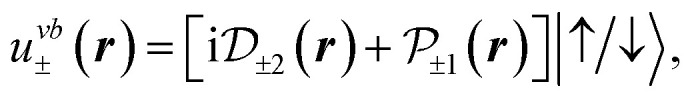
10d
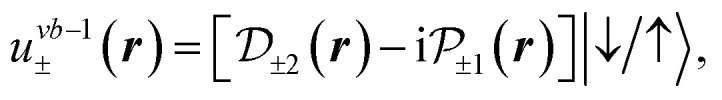
Here the functions 
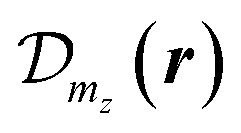
 and 
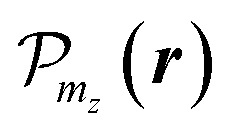
 (*m*_*z*_ = 0, ±1, ±2) denote the Bloch amplitudes formed by d and p atomic orbitals at metal and chalcogen atoms respectively. This form of wavefunctions assumes that the orbital part is even along the *z* axis, so 
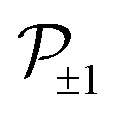
 orbitals have the same phase at the two chalcogen atoms in the same unit cell. The phases of the functions are chosen to form the standard basis of the corresponding representation of the *D*_3h_ group and to match the time reversal symmetry. In the ESI† we consider a more sophisticated tight binding model, which accounts for the mixing between spin-up and spin-down states.

Each pair of functions (10a)–(10d) forms the basis of an irreducible representation of the group *D*_3h_, and the two corresponding functions belong to the conjugated irreducible representations of the group *C*_3h_, in agreement with eqn (5). Note that in ref. 31, where the hyperfine interaction was studied using first principles, the expression for *u*^*vb*^_±_ was different, which led to some wrong results.

In the vicinity of the *n*th nucleus the orbital wavefunction has the form11
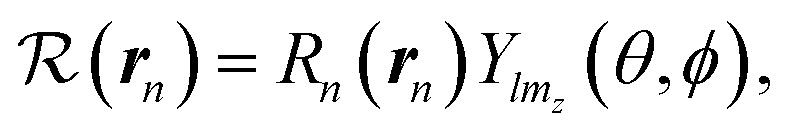
where the spherical harmonics *Y*_*lm*_*z*__(*θ*,*ϕ*) with *l* = 1, 2 correspond to the functions 
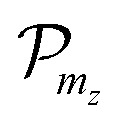
 and 
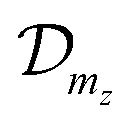
, respectively. The radial parts of the wavefunctions in eqn (11) are normalized as12
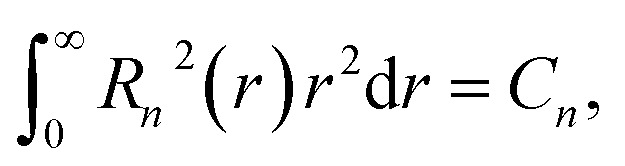
where *C*_*n*_ is the probability to find an electron at the corresponding atom.

Taking into account the explicit form of the wave functions, eqn (10), additional restrictions can be obtained for the hyperfine interaction tensors. Indeed, the Hamiltonian (9) cannot change the total electron angular momentum *f*_*z*_ = *m*_*z*_ + *s*_*z*_ by more than 1.‡‡The last term in eqn (9) being a part of the dipole–dipole interaction can change the electron orbital momentum *m*_*z*_ by ±2, but simultaneously the electron spin *s*_*z*_ changes by ∓1, so the total angular momentum *f*_*z*_ cannot be changed by more than 1, in agreement with the spherical symmetry of the Hamiltonian (9). The representation Γ_9_ corresponds to *f*_*z*_ = ±3/2, so these states cannot be mixed by the hyperfine interaction. In this case the components *A*_*xx*_ and *A*_*yy*_ vanish, in agreement with the general symmetry arguments. Additionally, the wavefunctions at the metal atoms in the upper valence band, *vb*, have the total angular momenta *f*_*z*_ = ±5/2 and hence *A*_*xx*_ = *A*_*yy*_ = 0 in this case (see Table 2). As a result the hyperfine interaction in the upper valence band is purely of the Ising type.

The calculation of the matrix elements of the Hamiltonian (9) with the wavefunctions described by eqn (10) and (11) yields the relationship between the in-plane and out-of-plane components of the hyperfine interaction tensors. These results are summarized in the last three columns in Tables 2 and 3. One can see that |*A*_*zz*_| = 2|*A*_*xx*_|, whenever *A*_*xx*_ is nonzero. The absolute values of the hyperfine interaction constants in Tables 2 and 3 are determined by13
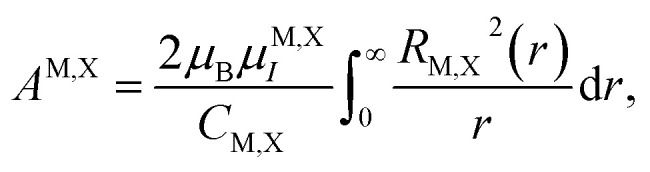
where M and X stand for the metal and chalcogen atoms, respectively. Importantly, the sign of *A*^M,X^ coincides with the sign of *μ*^M,X^_*I*_. From Tables 2 and 3, one can see that the sign of *A*_*zz*_ can be both positive and negative in different bands.

The noncollinear term ∝*τ*_*y*_Δ*I*_*z*_ in eqn (8) vanishes in the model described above. In the ESI† we consider the 22 band tight binding model,^37^ which takes into account all p states at both chalcogen atoms and all d states at metal atoms in the unit cell. This model describes weak mixing between spin-up and spin-down states, so the two Bloch wavefunctions in *K*_±_ valleys in the bands *cb* + 1 and *vb* at the chalcogen atoms above/below the (*xy*) plane have the form:14a
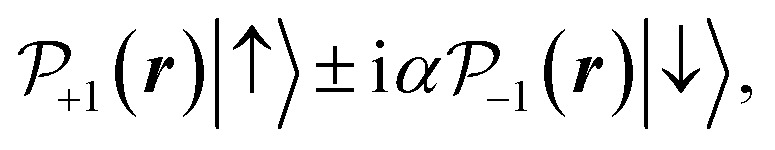
14b
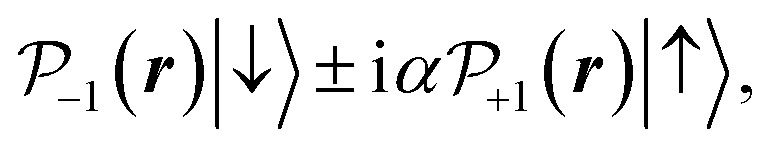
where ± sign corresponds to the two chalcogen atoms. These functions belong to the Γ_9_ representation, and it follows from the time reversal symmetry that the parameter *α* is real. A similar calculation of the matrix elements of the Hamiltonian (9) in the first order in a small parameter *α* shows that the off diagonal component of the hyperfine interaction tensor is15
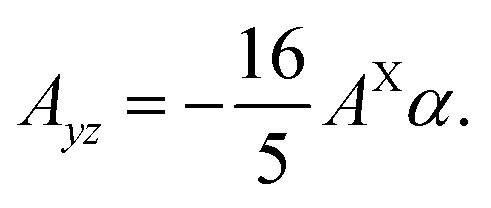
The tight binding model yields the result *α* = −0.08 in the band *cb* + 1, so the noncollinear term in the hyperfine interaction tensor is approximately 6 times smaller than the collinear one. In the band *vb* the calculation yields *α* = 0, so the hyperfine interaction in this band is purely of the Ising type.

Interestingly the optical activity of the “dark” excitons in tungsten based TMDs in *z* polarization^42^ is also related to the mixing of spin-up and spin-down states. Therefore the dipole moment in *z* polarization is proportional to the parameter *α*, but it also requires the overlap between 
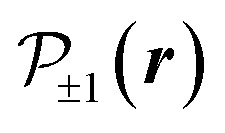
 orbitals at the two chalcogen atoms.

## 4. Discussion of the physical effects

In this section we provide the estimates for the hyperfine interaction constants, and describe and discuss the following physical effects: spin–valley polarization relaxation, dynamic nuclear spin polarization, dragging of the quantum dot resonance frequency and formation of the nuclear spin polaron.

First of all we note that the relationships obtained in Sec. 2 are strict and follow only from the symmetry analysis. Therefore the same results can also be applied to any substitutional impurity in TMD MLs.

In TMDs not all the metal and chalcogen isotopes have nonzero nuclear spins. The ones with nonzero nuclear spins are listed in Table 4 together with their abundances (*ν*) and spins (*I*). One can see that less than a half of atoms of each type have nonzero spins.

**Table 4:** Properties of the isotopes of metal and chalcogen free atoms with nonzero spin: mass number (*M*), abundance (*ν*), spin (*I*) and hyperfine interaction constant (*A*)

	*M*	*ν* (%)	*I*	*A* (μeV)
Mo	95	15.92	5/2	−0.57
	97	9.55	5/2	
W	183	14.31	1/2	0.64
S	33	0.76	3/2	0.75
Se	77	7.63	1/2	3.9
Te	123	0.89	1/2	−8.3
	125	7.07	1/2	

The values of the hyperfine interaction constants (*A*) can be calculated using atomistic approaches, for example DFT.^31,48,49^ The orbitals 
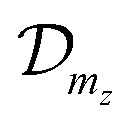
 at molybdenum and tungsten are related mainly to 4d and 5d atomic orbitals, respectively, while the orbitals 
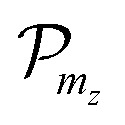
 are related to 3p, 4p and 5p atomic orbitals at sulfur, selenium and tellurium, respectively. The estimations for the hyperfine coupling constants can be obtained from the corresponding values known for the free atoms.^50^ They are given in the last column in Table 4. We note that the straightforward calculation of the integrals in eqn (13) for the Slater orbitals^51^ with the effective screening constants^52^ yields the values an order of magnitude smaller.

From Table 4 one can see that the hyperfine interaction is stronger for chalcogen atoms than for the metal atoms. It is related to the fact that p orbitals correspond to the smaller angular momentum and are more localized at the nuclei than d orbitals. Moreover, as one could expect, separately for metal and chalcogen atoms, the heavier is the atom the stronger is the hyperfine interaction. We note that the spin–orbit splitting of the conduction and valence bands in TMD MLs qualitatively obeys the same rules, which is related to the common relativistic origin of the two effects.

It is instructive to compare the hyperfine interaction parameters with those in the well studied semiconductor GaAs. In GaAs the spin–orbit splitting of the valence band is about 330 meV,^53^ which approximately equals to the splitting of the two uppermost valence bands in TMD MLs. The hyperfine interaction constants in the valence band of GaAs are of the order of 10 μeV,^54,55^ which is comparable to those in TMDs. By contrast, the hyperfine interaction in the conduction band of GaAs is an order of magnitude stronger due to the *s* type of the Bloch amplitudes.^56,57^ Hence the hyperfine interaction of electrons in TMD MLs is much weaker than in GaAs.

The most important effect related to the hyperfine interaction is the valley polarization relaxation. In large magnetic fields the loss of the polarization is dominated by the electron phonon interaction, but this mechanism predicts infinite spin relaxation time in zero magnetic field.^36^ In fact the polarization relaxation in small magnetic fields is related to the hyperfine interaction. The timescale related to the hyperfine interaction can be estimated as^58^16
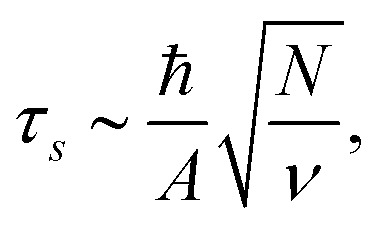
where *N* is the number of nuclei in the charge carrier localization volume. For the localization radius about 5 nm we find that *N* ∼ 10^3^ and *τ*_s_ ∼ 10–100 ns.

In order to describe the valley pseudospin dynamics quantitatively we use the model of Merkulov, Efros and Rosen.^58^ The polarization relaxation in zero magnetic field consists of two stages. In the first stage, the charge carrier spin precesses in the static fluctuation of the Overhauser field with the frequency **Ω**, while the nuclear spin dynamics can be neglected. The distribution function of the Larmor precession frequency has the form17
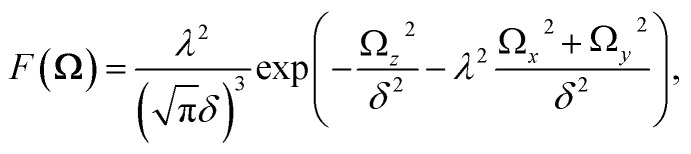
where the parameter 
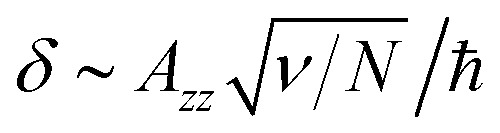
 describes the dispersion and18
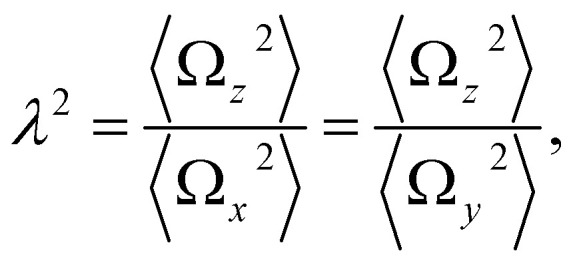
describes the anisotropy of the hyperfine interaction. Here the angular brackets denote the time or ensemble averaging. During the first stage the initial spin polarization along the *z* axis decreases on average by a factor *f*. In the second stage, the nuclear spin dynamics comes into play, and the rest of the polarization decays to zero on a parametrically longer timescale.

Let us consider the first stage in more detail. Provided that the valley pseudospin is initially oriented along the *z* direction, the dynamics of this component is described by

19 *τ*_*z*_(*t*) = *τ*_*z*_(0)[cos^2^ (*θ*) + sin^2^ (*θ*) cos (Ω*t*)],

where *θ* is an angle between **Ω** and *z* directions. The second term of this expression describes the pseudospin precession, while the first term corresponds to the component of ***τ*** parallel to **Ω**, which is a constant. The average of this equation over the distribution function (17) gives the observable average time dynamics of the valley polarization.

**Fig. 3:**
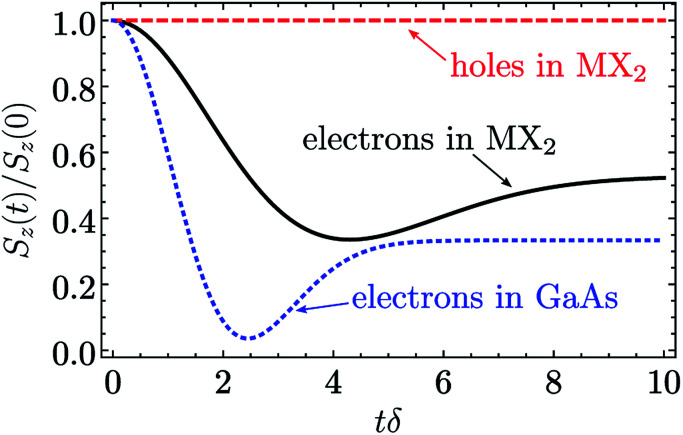
Polarization relaxation due to the hyperfine interaction. The spin relaxation of electrons in TMD MLs (black solid curve), holes in TMD MLs (red dashed curve) and electrons in typical GaAs quantum dots is described by the anisotropy parameter *λ* = 2, ∞ and 1 (see eqn (17) and (19)), respectively.

Fig. 3 shows this dynamics. The black curve corresponds to the spin relaxation of localized electrons in TMD MLs, where *λ* = 2 (see Tables 2 and 3). The spin polarization decreases due to the spin precession in the random nuclear field. However, the component of the spin parallel to the nuclear field does not precess, so the fraction *f* of the initial spin polarization is conserved. For comparison the blue curve corresponds to the electrons in typical GaAs quantum dots, where the hyperfine interaction is isotropic (*λ* = 1). Compared to this case the spin polarization of electrons in TMD ML quantum dots decays slower, and the larger part of spin polarization is preserved during the first stage. Finally, the red curve corresponds to the localized holes in TMD MLs. It follows from Tables 2 and 3 that in this case Ω_*x*_ = Ω_*y*_ = 0, so the spin polarization is constant, and it does not decay due to the hyperfine interaction on the time scale under study.

The fraction of the initial spin polarization conserved during the first stage can be calculated analytically:^30^20
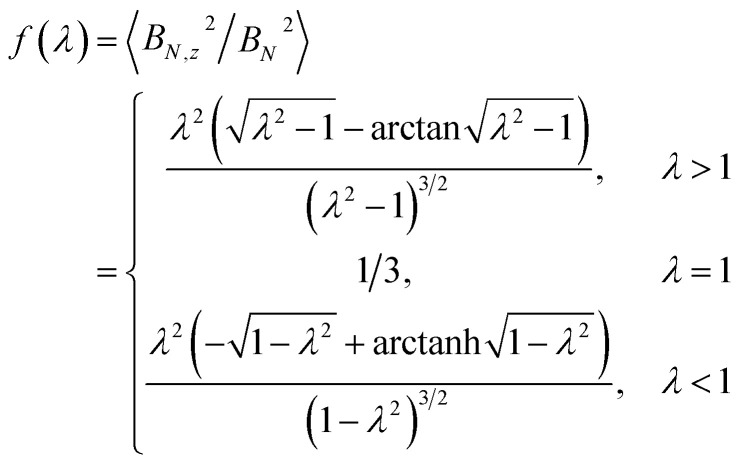
One can see that in the case of the isotropic hyperfine interaction *f*(1) = 1/3. In the lower conduction band *λ* = 2, and21
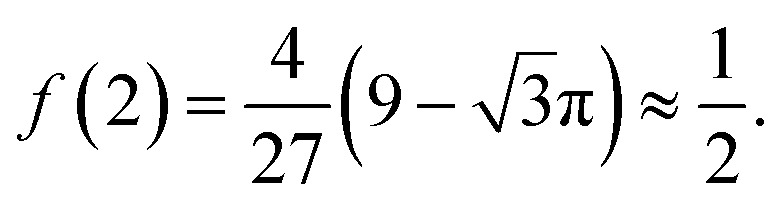
Therefore, the valley polarization of localized charge carriers in this band decreases approximately two times in a few tens of nanoseconds (see Fig. 3), before decaying to zero on a longer time scale. A small admixture of *s*-type orbitals at metal atoms^3^ can slightly change this ratio.

The most interesting situation in TMD MLs takes place in the upper valence band. Here, as follows from Tables 2 and 3, *λ* = ∞ and *f* = 1. This describes the situation, when the relaxation of the spin *z* component is absent because of the Ising type of the hyperfine interaction. For chalcogen atoms this is the symmetry requirement, while for metal atoms this results from 
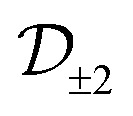
 atomic orbitals, which cannot be mixed by the hyperfine interaction Hamiltonian (9). Therefore, one should expect longest valley coherence times for localized holes in TMD MLs.

On long timescales the valley relaxation of localized holes can be related to (i) two phonon processes,^59–61^ (ii) single phonon processes in combination with the hyperfine interaction,^62^ or (iii) mixing of the energy degenerate states by the localization potential.^36,63,64^ In the latter case, the localization potential should be atomically sharp, *e.g.* an impurity. Otherwise, the degree of mixing of the two valleys is proportional to the ratio *κ* of the lattice constant and the localization length. This situation is similar to the one described above with an effective degree of the hyperfine interaction anisotropy *λ*_eff_ ∼ 1/*κ*.

Apart from the spin relaxation the hyperfine interaction gives rise also to the effect of dynamic nuclear spin polarization.^65^ This effect consists in the transfer of spin polarization from the charge carriers to nuclei. The dynamic nuclear spin polarization is inefficient in the upper valence band of TMD MLs, but in all the other bands it can be significant.

Dynamic nuclear spin polarization is most pronounced under excitation by circularly polarized light. It orients valley pseudospin along the *z* axis, and this polarization is transferred to nuclear spins. Uniform nuclear spin polarization creates an Overhauser field, which splits the optical resonance in TMD ML quantum dots, and this slitting can be observed experimentally.

The observation of the helical nuclear spin polarization requires in-plane valley pseudospin polarization. We note that the transverse magnetic field does not mix the free states in the different valleys because of the translational invariance. For charge carriers and charged complexes, however, this restriction is removed. For example, in symmetric quantum dots with the symmetry *D*_3h_ and with the center at the metal atom the two ground electron states belong to the Γ_7_ representation, see Table 2. The direct product Γ_7_ ⊗ Γ_7_ contains the representation Γ_5_, which corresponds to the in-plane magnetic field. As a result the magnetic field can lead either to thermal in-plane pseudospin polarization or to the rotation of out-of-plane polarization to the (*xy*) plane. The in-plane valley pseudospin leads to electron-nuclear spin flip flops and to chiral dynamic nuclear spin polarization. The chiral nuclear spin polarization manifests itself, for example, similar to the transverse nuclear spin polarization in the Hanle effect.^65^

An example of nuclear spin polarization distribution for ***τ***‖***x*** in the lowest conduction band of molybdenum based TMD MLs is shown in Fig. 1. Notably, the distribution of nuclear spin polarization is nonuniform. It is described by the phases *ϕ*_*n*_ in eqn (7) and looks like rotating nuclear spins. So we call it the “helical” structure of nuclear spin polarization. Despite the nuclear spin ordering a macroscopic nuclear spin polarization is absent, which is analogous to an antiferromagnetic spin state.

The helical hyperfine interaction is essentially based on valley degeneracy in contrast to many other effects. In fact, it requires two ingredients: (i) valley degeneracy and (ii) strong spin–orbit interaction, which lifts the spin degeneracy in each valley. For example, this effect cannot take place in usual GaAs-based structures, where the valley degeneracy is absent. In SiGe quantum wells the spin–orbit interaction also does not lead to the spin slitting of the valleys,^66^ and in this case the intervalley hyperfine interaction is hidden by the intravalley interaction. Thus TMD MLs are the most suitable structures for the observation of the helical hyperfine interaction. This observation is also facilitated by the direct band gap of these materials.

Interestingly the *D*_3h_ symmetry of TMD MLs allows for the noncollinear hyperfine interaction in the bands *vb* and *cb* + 1 with chalcogen atoms. Estimations made in the previous section show that *A*_*yz*_ in the band *cb* + 1 is only 6 times smaller than *A*_*zz*_. For comparison in GaAs quantum dots, the noncollinear term is two orders of magnitude smaller.^35^ The pronounced noncollinear hyperfine interaction in TMD MLs can lead to effective nuclear spin relaxation in a strong external perpendicular magnetic field (see the discussion above). Indeed, it allows flipping nuclear spins without changing the electron energy by the Zeeman energy. The same processes are also responsible for the “dragging” of the quantum dot resonance frequency by laser light.^67^ Based on Tables 2 and 3 we predict that this effect is particularly strong for quantum dots based on tungsten dichalcogenides, where the lowest conduction band corresponds to *cb* + 1 in our notations.

Finally, we note that in the case of charge carrier localization in a two dimensional structure, the many-body nuclear spin effects such as nuclear spin self-polarization^68,69^ and formation of nuclear spin polaron^70,71^ can be pronounced. The nuclear spin polaron can also reveal the helical structure of the hyperfine interaction. These effects can be used to additionally increase the valley pseudospin relaxation time and to realize robust control of its orientation, similarly to magnetic skyrmions.^72,73^

## 5. Conclusions

Using a symmetry and microscopic analysis we obtained explicit expressions for hyperfine interaction tensors in TMD MLs (Tables 2 and 3). Based on the form of the effective hyperfine interaction Hamiltonian we predict the following effects: (i) The valley polarization of localized electrons decays approximately two times in a few tens of nanoseconds. (ii) The polarization of localized holes decays parametrically slower than that of electrons. (iii) The hyperfine interaction between electron spins in tungsten dichalcogenide MLs is strongly noncollinear. (iv) The in-plane dynamic nuclear spin polarization is helical, *i.e.* nonuniform in space, which is a direct manifestation of spin–valley locking.

## Conflicts of interest

There are no conflicts to declare.

## Supplementary Material

NA-001-C8NA00360B-s001
