# Supplementary material for: Hyperfine interaction in atomically thin transition metal dichalcogenides
Source: Nanoscale Adv. 2019 May 13;1(7):2624–32. doi: 10.1039/c8na00360b (PMC9417057; doi:10.1039/c8na00360b)
Supplement: NA-001-C8NA00360B-s001 [file NA-001-C8NA00360B-s001.pdf]

Electronic Supplementary Material (ESI) for Nanoscale Advances.
This journal is © The Royal Society of Chemistry 2019

Electronic Supplementary Information to "Hyperfine interaction in atomically thin transition metal dichalcogenides"

I. D. Avdeev[1] and D. S. Smirnov[1,*]
[1]*Ioffe Institute, 194021 St. Petersburg, Russia*

CONTENTS

S1. Summary of TMD ML structure 1

S2. Shift of point symmetry group transformations center 1

S3. Pseudospin operators 2

S4. Tight binding model 3

References 3

S1. SUMMARY OF TMD ML STRUCTURE

A single TMD ML has the honeycomb lattice with the two dimensional translation vectors $\boldsymbol{a}_1 = a_0(1,0)$ and $\boldsymbol{a}_2 = a_0(1/2, \sqrt{3}/2)$, where a_0 is the lattice constant. The choice of the coordinate frame is described in the main text and is shown in Fig. 1. The metal atoms lie within the ML plane (xy), while the chalcogen atoms are shifted from the plane along and opposite to z axis. The position of the metal atom in the elementary cell is $\boldsymbol{\tau} = a_0/2(1, 1/\sqrt{3})$, and the two chalcogen atoms are located above and below the point $-\boldsymbol{\tau}$.

The reciprocal lattice is determined by the basis vectors $\boldsymbol{b}_1 = 2\pi/a_0(1, -1/\sqrt{3})$ and $\boldsymbol{b}_2 = 2\pi/a_0(0, 2/\sqrt{3})$. The extremes of the conduction and the valence bands are located at the two inequivalent corners of the hexagonal Brillouin zone, i.e. in $\boldsymbol{K}_\pm = 2\pi/a_0(\pm 2/3, 0)$ valleys.

S2. SHIFT OF POINT SYMMETRY GROUP TRANSFORMATIONS CENTER

For the symmetry analysis, described in the main text, it is necessary to find the irreducible representations of the electronic states for the choices of the point symmetry origins at the points $\pm\boldsymbol{\tau}$. Here we present a formalism, which solves this problem. It can also be easily generalized for the other problems in physics of multivalley semiconductors.

Let us consider a spatial group $G_{\boldsymbol{k}}$ of the wave vector $\boldsymbol{k} = \boldsymbol{K}_\pm$. Further, let us assume, that this group is symmorphic, as in the case of TMD MLs. Let T be the translation subgroup. Then the quotient group $P_{\boldsymbol{k}} = G_{\boldsymbol{k}}/T$, being the set of rotations $\{R\}$, is the corresponding point group. By definition, for each R [S1]

$$R\,\boldsymbol{k} = \boldsymbol{k} + \boldsymbol{b}\,, \tag{S1}$$

where $\boldsymbol{b}$ is a reciprocal lattice vector (or zero vector).

Now consider a set of the Bloch wave functions $\{\psi_n\} = \{e^{i\boldsymbol{k}\boldsymbol{r}} u_n(\boldsymbol{r})\}$, with $u_n(\boldsymbol{r})$ being the Bloch amplitude, that forms the basis of an irreducible representation D of the point group $P_{\boldsymbol{k}}$. Note, that in case of TMD ML, all the irreducible representations are one dimensional, but here we consider a general situation. The wave functions transform under the action of R as

$$R\,\psi_n = \sum_{n'} \psi_{n'}\, D_{n'n}(R)\,. \tag{S2}$$

where $\hat{D}(R)$ are the matrices of the irreducible representation D.

We introduce the point group $P'_{\boldsymbol{k}}$ with the transformations center shifted by a vector $\boldsymbol{t}$ as

$$P'_{\boldsymbol{k}} = \hat{\boldsymbol{t}}\, P_{\boldsymbol{k}}\, \hat{\boldsymbol{t}}^{-1}, \tag{S3}$$

where $\hat{\boldsymbol{t}}$ denotes the translation operator. The nontrivial case corresponds to $\boldsymbol{t}$ being not a translation vector. The elements g' of $P'_{\boldsymbol{k}}$ have the form

$$g' = (R|\boldsymbol{t} - R\,\boldsymbol{t}), \tag{S4}$$

being the rotation R with the subsequent shift by the vector $\boldsymbol{t} - R\,\boldsymbol{t}$.

For TMD ML for $\boldsymbol{t} = \pm\boldsymbol{\tau}$, the vector $\boldsymbol{t} - R\,\boldsymbol{t}$ is a translation vector for each R, because there are only one metal atom and one pair of chalcogen atoms in the primitive cell. Once this condition is satisfied, one can show that the set $\{e^{-i\boldsymbol{k}(\boldsymbol{t}-R\boldsymbol{t})}\}$ forms a one dimensional representation $D_{\boldsymbol{t}}$ of the group $P_{\boldsymbol{k}}$.

To prove this statement let us consider the two rotations $R_1, R_2 \in P_{\boldsymbol{k}}$ and the corresponding translation vectors

$$\boldsymbol{\alpha}_{1,2} = \boldsymbol{t} - R_{1,2}\boldsymbol{t}\,. \tag{S5}$$

The scalar product is invariant under rotations $\boldsymbol{k}R_1\boldsymbol{\alpha}_2 = \left(R_1^{-1}\boldsymbol{k}\right)\boldsymbol{\alpha}_2$, so

$$\mathrm{e}^{i\boldsymbol{k}(\boldsymbol{\alpha}_2 - R_1\boldsymbol{\alpha}_2)} = 1$$

by the definition of $P_{\boldsymbol{k}}$, see Eq. (S1). From this relation, using Eq. (S5), we readily obtain

$$e^{-i\boldsymbol{k}(\boldsymbol{t}-R_1\boldsymbol{t})} e^{-i\boldsymbol{k}(\boldsymbol{t}-R_2\boldsymbol{t})} = \mathrm{e}^{-\mathrm{i}\boldsymbol{k}(\boldsymbol{t}-R_1R_2\boldsymbol{t})},$$

TABLE I. From left to right: the bands; C_{3h} irreducible representations of the electronic states in $\boldsymbol{K}_\mp$ valley with the transformations center at the midpoint of the hexagon and at a metal atom (M); the irreducible representations in D_{3h} group and the basis functions (see Table III); the form of the hyperfine interaction Hamiltonian.

band	C_{3h}		C_{3h}, M		D_{3h}, M			H_{hf}
	$\boldsymbol{K}_-$	$\boldsymbol{K}_+$	$\boldsymbol{K}_-$	$\boldsymbol{K}_+$	irrep	$\boldsymbol{K}_-$	$\boldsymbol{K}_+$	
$cb+1$	Γ_{10}	Γ_9	Γ_7	Γ_8	Γ_7	ψ_2^7	ψ_1^7	H_-
cb	Γ_{12}	Γ_{11}	Γ_8	Γ_7	Γ_7	ψ_1^7	ψ_2^7	H_+
vb	Γ_8	Γ_7	Γ_9	Γ_{10}	Γ_8	ψ_2^8	ψ_1^8	H_-
$vb-1$	Γ_7	Γ_8	Γ_{11}	Γ_{12}	Γ_9	ψ_1^9	ψ_2^9	H_z

TABLE II. From left to right: the bands; C_{3h} irreducible representations of the electronic states in $\boldsymbol{K}_\mp$ valley with the transformations center at the midpoint of the hexagon and between neighboring chalcogen atoms (X); the irreducible representations in D_{3h} group and the basis functions (see table III); the form of the hyperfine interaction Hamiltonian.

band	C_{3h}		C_{3h}, X		D_{3h}, X			H_{hf}
	$\boldsymbol{K}_-$	$\boldsymbol{K}_+$	$\boldsymbol{K}_-$	$\boldsymbol{K}_+$	irrep	$\boldsymbol{K}_-$	$\boldsymbol{K}_+$	
$cb+1$	Γ_{10}	Γ_9	Γ_{11}	Γ_{12}	Γ_9	ψ_1^9	ψ_2^9	H_z
cb	Γ_{12}	Γ_{11}	Γ_9	Γ_{10}	Γ_8	ψ_2^8	ψ_1^8	H_-
vb	Γ_8	Γ_7	Γ_{12}	Γ_{11}	Γ_9	ψ_2^9	ψ_1^9	H_z
$vb-1$	Γ_7	Γ_8	Γ_{10}	Γ_9	Γ_8	ψ_1^8	ψ_2^8	H_+

which proves the multiplication rule, and thus the statement.

Therefore, the set of the functions $\{\psi_n\}$, which transforms according to Eq. (S2), is the basis of a representation

$$D' = D \otimes D_{\boldsymbol{t}}\ , \tag{S6}$$

of the point group $P'_{\boldsymbol{k}}$, where $D_{\boldsymbol{t}}(R) = e^{-i\boldsymbol{k}(\boldsymbol{t}-R\boldsymbol{t})}$.

In the $\boldsymbol{K}_-$ ($\boldsymbol{K}_+$) valley of a TMD ML, $D_{\boldsymbol{\tau}}$ is the $\Gamma_{2(3)}$ representation and $D_{-\boldsymbol{\tau}}$ is the representation $\Gamma_{3(2)}$ of the C_{3h} point group. The multiplication of the representations allows us to find the irreducible representations of the wave functions with different centers of transformations. The representations of D_{3h} group are summarized in the tables in the main text, while the representation of C_{3h} group are given in Tables I and II. The correspondence between them is established using the compatibility Table III.

S3. PSEUDOSPIN OPERATORS

Here we explicitly construct the pseudospin operators in the dyadic form, which act in the valley pseudospin 2×2 space. The bases of the representations are chosen in agreement with Ref. S2.

We denote the standard bases of Γ_7 (Γ_8) representation as $\boldsymbol{u} = (|1\rangle\,, |2\rangle) = (|-J\rangle\,, |+J\rangle)$ and $\boldsymbol{v} = (-\langle 2|\,, \langle 1|)$.

TABLE III. Compatibility chart of irreducible representations and basis functions for D_{3h} and C_{3h} point symmetry groups.

D_{3h}			C_{3h}	
Γ_7	ψ_1^7	$\lvert -1/2\rangle$	Γ_8	ψ_1^7
	ψ_2^7	$\lvert +1/2\rangle$	Γ_7	ψ_2^7
Γ_8	ψ_1^8	$\lvert -1/2\rangle\, zS_z$	Γ_{10}	ψ_1^8
	ψ_2^8	$\lvert +1/2\rangle\, zS_z$	Γ_9	ψ_2^8
Γ_9	ψ_1^9	$\lvert -3/2\rangle$	Γ_{11}	ψ_1^9
	ψ_2^9	$\lvert +3/2\rangle$	Γ_{12}	ψ_2^9

These two bases can be obtain one from another performing the time reversal with the Hermitian conjugation. Thus they transform in the same way for all symmetry operations g:

$$g\,\boldsymbol{u} = \boldsymbol{u}\,\hat{D}(g), \tag{S7a}$$

$$g\,\boldsymbol{v} = \boldsymbol{v}\,\hat{D}(g). \tag{S7b}$$

Using the coupling coefficients one can easily find operators belonging to Γ_2 representation

$$\hat{\tau}^{(2)} = \frac{i}{\sqrt{2}}(|1\rangle\langle 1| - |2\rangle\langle 2|) \propto \hat{\tau}_z\ , \tag{S8}$$

and Γ_5 representation

$$\begin{aligned} \hat{\tau}_1^{(5)} &= -\,|1\rangle\langle 2| \propto \quad (\hat{\tau}_x - i\hat{\tau}_y)\ , \\ \hat{\tau}_2^{(5)} &= \quad |2\rangle\langle 1| \propto -(\hat{\tau}_x + i\hat{\tau}_y)\ . \end{aligned} \tag{S9}$$

They are proportional to the components of the valley pseudospin operator $\hat{\boldsymbol{\tau}}$, because they transform in the same way. The intervalley hyperfine interaction Hamiltonian can be written in a matrix form as

$$H_\pm = A_\perp(I_x\tau_x \pm I_y\tau_y) + A_\parallel I_z\tau_z\ , \tag{S10}$$

where $A_\perp$ and $A_\parallel$ are the constants and the different signs correspond to the different order of the basis functions $(|1\rangle\,, |2\rangle)$ or $(|2\rangle\,, |1\rangle)$. The signs are explicitly written in the last columns of Tables I and II.

For Γ_9 states the pseudovector operator $\hat{\tau}_z$, belonging to Γ_2, is given by Eq. (S8), while the Γ_3 operator has the form

$$\hat{\tau}^{(3)} = -\frac{1}{2}\,(|1\rangle\langle 2| - |2\rangle\langle 1|) \propto \hat{\tau}_y\ . \tag{S11}$$

The symmetry allowed hyperfine interaction Hamiltonian is

$$\hat{H}_z = A_\parallel\tau_z I_z + A_{yz}\tau_y\Delta I_z\ . \tag{S12}$$

The bands and atoms relevant for this case are also given in Tables I and II in the last columns.

S4. TIGHT BINDING MODEL

We complement the symmetry analysis described in the main text with calculations performed in the 22 band tight binding model from Ref. S3. The model uses five atomic orbitals at metal atom D_{m_z} ($m_z = -2\dots2$), and six atomic orbitals at chalcogens $P^{up}_{m_z} = P_{m_z}(\boldsymbol{r}-\boldsymbol{h})$ and $P^{down}_{m_z} = P_{m_z}(\boldsymbol{r}+\boldsymbol{h})$ ($m_z = -1,0,1$), where $\boldsymbol{h} = (0,0,h)$ is the chalcogens displacement from the TMD plane. The model also includes on-site spin orbit interaction, which is included following Ref. S4.

The calculation yields the following Bloch amplitudes in $\boldsymbol{K}_\pm$ valleys:

$$u^{cb+1}_\pm = \pm 0.9138\mathrm{i}D_0 \left|\downarrow / \uparrow\right\rangle \pm 0.4039P_{\mp1}\left|\downarrow / \uparrow\right\rangle \mp 0.0269\mathrm{i}D_{\mp1}\left|\uparrow / \downarrow\right\rangle \mp 0.0322\mathrm{i}\Delta P_{\pm1}\left|\uparrow / \downarrow\right\rangle, \tag{S13a}$$

$$u^{cb}_\pm = \pm 0.9155D_0\left|\uparrow / \downarrow\right\rangle \mp 0.4012\mathrm{i}P_{\mp1}\left|\uparrow / \downarrow\right\rangle \mp 0.014D_{\pm1}\left|\downarrow / \uparrow\right\rangle \mp 0.028\mathrm{i}P_0\left|\downarrow / \uparrow\right\rangle, \tag{S13b}$$

$$u^{vb}_\pm = 0.899\mathrm{i}D_{\pm2}\left|\uparrow / \downarrow\right\rangle + 0.438P_{\pm1}\left|\uparrow / \downarrow\right\rangle, \tag{S13c}$$

$$u^{vb-1}_\pm = 0.8934D_{\pm2}\left|\downarrow / \uparrow\right\rangle - 0.4484\mathrm{i}P_{\pm1}\left|\downarrow / \uparrow\right\rangle + 0.0045D_{\pm1}\left|\uparrow / \downarrow\right\rangle - 0.0299\mathrm{i}P_0\left|\uparrow / \downarrow\right\rangle. \tag{S13d}$$

Here we introduced $P_{m_z} = (P^{up}_{m_z} + P^{down}_{m_z})/\sqrt{2}$ and $\Delta P_{m_z} = (P^{up}_{m_z} - P^{down}_{m_z})/\sqrt{2}$. Phases of the wavefunctions above are chosen to satisfy the two rules: (i) The functions $u^m_\pm$ form the standard basis of the corresponding irreducible representation of D_{3h} group with the center of transformations at the center of hexagon [S2]. (ii) The function u^m_+ is obtained from u^m_- by the time reversal symmetry, which assumes the following transformation of spinors:

$$\left|\uparrow\right\rangle \to \left|\downarrow\right\rangle, \qquad \left|\downarrow\right\rangle \to -\left|\uparrow\right\rangle. \tag{S14}$$

The wavefunctions $u^{cb+1}_\pm$ correspond to the anisotropy parameter $\alpha \approx 0.08$, which describes the noncollinear term $A_{yz}\tau_y\Delta I_z$ in the main text. As a result, in this model the noncollinear interaction is only 6 times weaker, than the collinear one. At the same time this model does not predict any corrections to the purely Ising type hyperfine interaction in the upper valence band (vb).

Finally, we note, that in W (tungsten) based TMDs the order of cb and $cb+1$ bands is inverted. In this case the ground exciton state is dark in xy polarizations In z polarization it is optically active [S5]. The corresponding dipole matrix element, is proportional to the overlap of $P^{up}_{\pm1}$ and $P^{down}_{\pm1}$ orbitals and the anisotropy parameter α.

* smirnov@mail.ioffe.ru

[S1] G. L. Bir and G. E. Pikus, *Symmetry and deformation effects in semiconductors (in Russian)* (Moscow (Nauka), 1972).
[S2] G. F. Koster, R. G. Wheeler, J. O. Dimmock, and H. Statz, *Properties of the thirty-two point groups* (MIT Press, 1963).
[S3] S. Fang, D. R. Kuate, S. N. Shirodkar, S. Lieu, G. A. Tritsaris, and E. Kaxiras, Phys. Rev. B **92**, 205108 (2015).
[S4] D. J. Chadi, Phys. Rev. B **16**, 790 (1977).
[S5] G. Wang, C. Robert, M. M. Glazov, F. Cadiz, E. Courtade, T. Amand, D. Lagarde, T. Taniguchi, K. Watanabe, B. Urbaszek, and X. Marie, Phys. Rev. Lett. **119**, 047401 (2017).
